# Sacrococcygeal Meningocele Antenatally Misdiagnosed as Teratoma: A Case Report

**DOI:** 10.7759/cureus.36485

**Published:** 2023-03-21

**Authors:** Hani A Alnajjar, Shaden S Almousa, Abdulelah S Almousa, Muhannad M Al Wadany

**Affiliations:** 1 Department of Neurosurgery, King Faisal University, Hofuf, SAU

**Keywords:** case report, antenatal ultrasound, teratoma, meningocele, sacrococcygeal mass

## Abstract

Sacrococcygeal masses encompass a diverse range of pathologies. Prenatal ultrasound facilitates early detection of congenital sacrococcygeal masses. We present the case of a newborn of a 22-year-old woman who was identified to have a sacrococcygeal mass by prenatal ultrasound that was initially diagnosed as sacrococcygeal teratoma. On examination after delivery, a large midline mass in the sacrococcygeal region was observed, which was globular in shape and had smooth, thin skin with bluish discoloration. Magnetic resonance imaging revealed a cystic lesion that protruded through a caudal sacral defect, consistent with a sacrococcygeal meningocele. The patient underwent surgical repair of the meningocele without any intraoperative complications and had preserved motor function in the lower extremities after the procedure. This case underscores the challenge of distinguishing sacrococcygeal teratoma from meningocele based on clinical presentation and prenatal ultrasound findings. An accurate preoperative diagnosis is essential for effective surgical planning.

## Introduction

Sacrococcygeal masses may arise from various etiologies such as congenital, traumatic, neoplastic, or inflammatory pathologies [1]. Congenital sacrococcygeal masses are commonly identified during the early stages of gestation due to advances in perinatal screening and the widespread use of ultrasound [2]. Meningocele is a rare congenital anomaly characterized by herniation of a spinal cerebrospinal fluid sac lined with leptomeninges, without neural tissue involvement. It is the simplest form of neural tube defect and accounts for 2.4% of spinal dysraphism cases [3]. The multifactorial etiology of neural tube defects involves genetic, environmental, and autoimmune factors. Genetic syndromes, pregestational diabetes mellitus, and maternal obesity have been associated with neural tube defects. However, most cases of isolated meningocele are related to folate deficiency [3]. In this report, we present a case of a newborn with an isolated sacrococcygeal meningocele initially diagnosed as a sacrococcygeal teratoma. The meningeal sac was large, arising from sacral defects and causing anterior displacement of the coccyx.

## Case presentation

**Figure 1:**
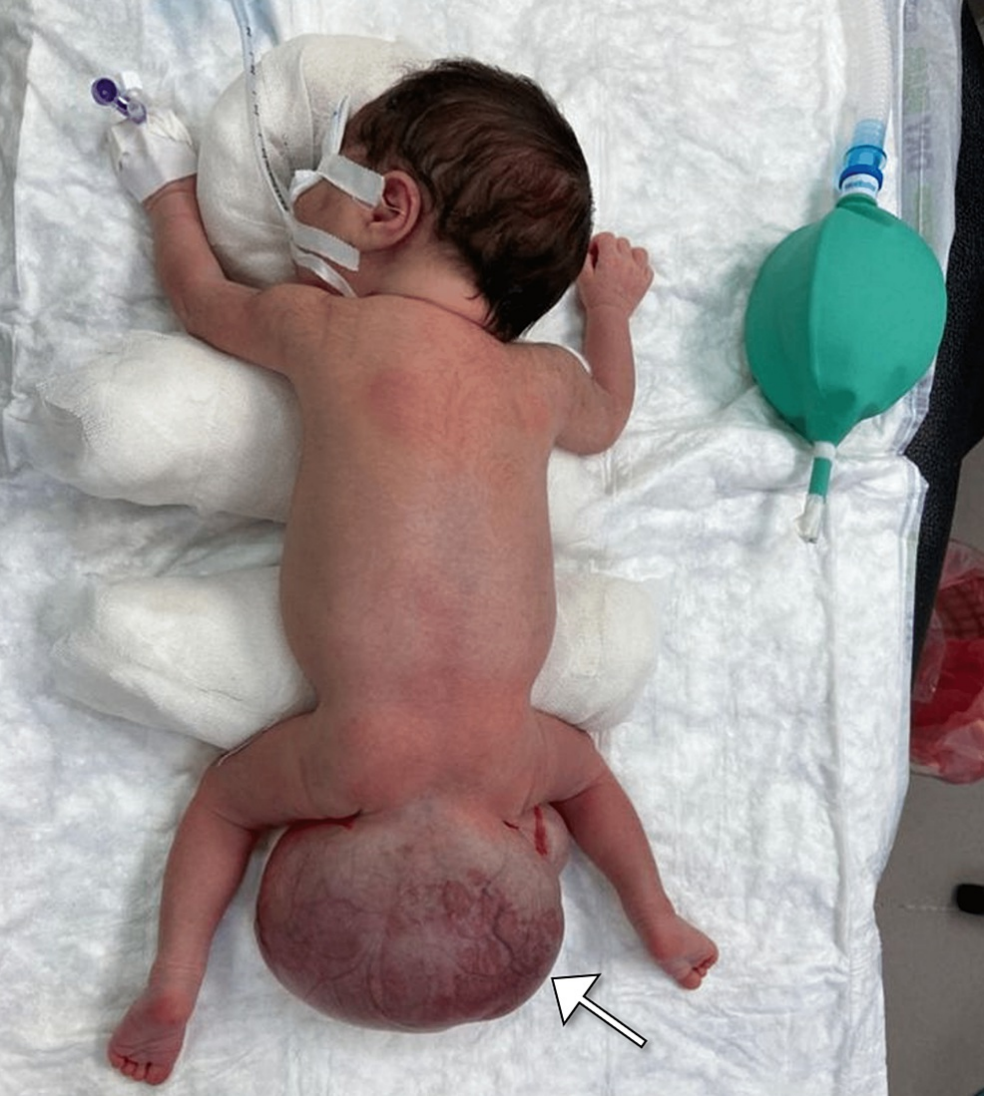
Photograph of the newborn demonstrating a large sacrococcygeal mass (arrow).

We describe the case of a female newborn delivered at 37 weeks gestation with a prenatal diagnosis of a sacrococcygeal mass. The mass was identified during the routine antenatal ultrasound examination in the first trimester and appeared as a complex mass with heterogeneous echogenicity in the sacrococcygeal region. The patient was delivered via cesarean section, and the mother had an unremarkable pregnancy history. Upon physical examination, a large midline mass in the sacrococcygeal region was observed. The mass had a globular shape, was covered by smooth and thin skin, and displayed bluish discoloration (Figure 1). The cardiovascular, respiratory, and neurological systems examinations were unremarkable, and laboratory investigations, including hematologic and biochemical parameters, were within normal limits.

**Figure 2:**
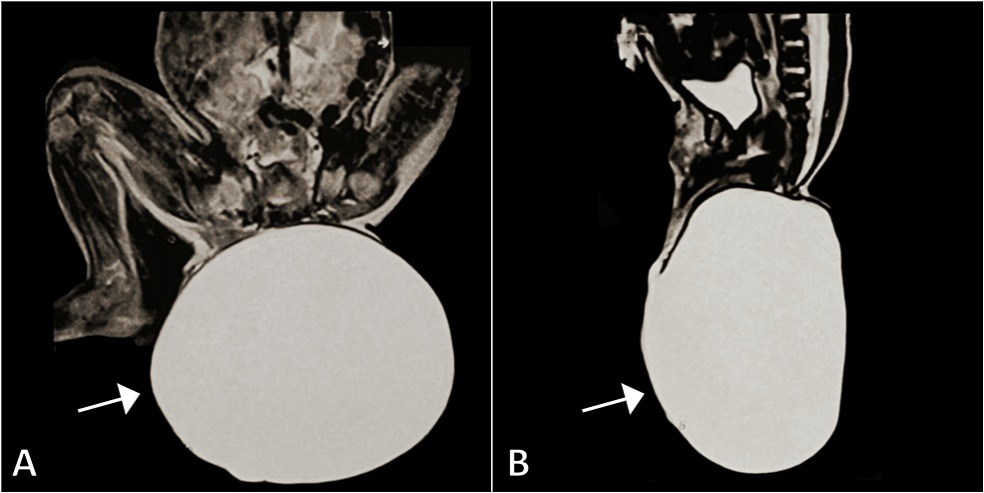
Coronal (A) and sagittal (B) MR images showing a large cystic sacrococcygeal lesion (arrows). MR: Magnetic resonance

To better diagnose and characterize the sacrococcygeal mass, magnetic resonance imaging (MRI) was performed. The results of the MRI showed an extradural cystic lesion without a solid component or enhancement. The lesion had a similar signal intensity to that of cerebrospinal fluid, with low signal intensity in T1-weighted images and high signal intensity in T2-weighted images. Additionally, it was protruding through a caudal sacral defect, and these findings were consistent with a sacrococcygeal meningocele (Figure 2).

After discussing the findings with the parents, it was decided that surgical repair of the meningocele would be performed. The surgical technique used in this case involved preoperative planning of the incision to ensure optimal cosmetic results, as well as preoperative marking of feeding vessels such as arteries and veins.

**Figure 3:**
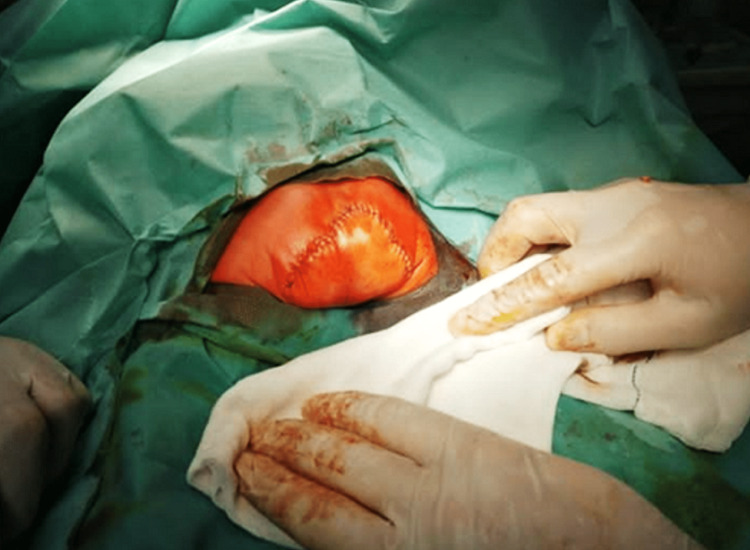
Postoperative photograph of the patient following successful removal of the sacrococcygeal mass.

During the surgery, the surgeon performed a delicate dissection of the meningeal sac, with careful attention to bleeding control, resulting in a total blood loss of 50 ml. Fortunately, no blood transfusion was needed. The herniated sac was large, had a thin vascular wall, was filled with cerebrospinal fluid, and did not contain any neural tissues. It was in direct contact with the rectum anteriorly and displaced the coccyx posteriorly. The perineum was divided and displaced laterally, and the sac was completely removed after securing both the urethra and rectum. The glucose level of the sac content was 55 mg/dl, which was comparable to that of cerebrospinal fluid. Primary closure of the perineum was then performed, followed by the closure of the incision edges and skin layers using absorbable stitching. The end result was a considerable cosmetic improvement of the buttocks, and remarkably, there was no need for a wound suction drain. The patient tolerated the procedure well, and no intraoperative complications were noted (Figure 3).

After the surgery, the patient exhibited preserved motor power in the lower extremities, and normal urination and bowel movement resumed. No evidence of sphincteric injury was observed. The patient was discharged after two days, in good general and neurological condition. It is worth noting that the baby was followed up for 1.5 years postoperatively, during which no neurological complications were observed.

## Discussion

The case presented highlights the complexity and challenges in the diagnosis and management of neural tube defects. Neural tube defects are a heterogeneous group of abnormalities that involve the central nervous system, with varying degrees of severity [3]. These defects can be broadly classified into two categories: anencephaly and spinal bifida. Spinal bifida is further classified into spina bifida occulta and spina bifida aperta, based on the presence or absence of associated abnormalities of the spinal cord or nerve roots [3].

The etiology of neural tube defects is multifactorial, with both genetic and environmental factors contributing to their development. Studies have shown an increased prevalence of neural tube defects among first-degree relatives of affected patients, indicating a genetic component to their pathogenesis [4]. However, environmental factors also play a crucial role in the development of these defects. Maternal obesity, diabetes, and hyperthermia during pregnancy are all associated with an increased risk of neural tube defects [4]. Additionally, folic acid supplementation has been shown to reduce the occurrence of these defects, highlighting the importance of maternal nutrition during fetal development.

In the present case, the initial clinical diagnosis of a sacrococcygeal teratoma highlights the challenges in differentiating between different types of sacrococcygeal masses. Sacrococcygeal teratoma is a rare tumor that arises from totipotent stem cells and is composed of two or three germ cell layers. It is considered the most common tumor in the neonatal period and can be diagnosed prenatally during an ultrasound examination [5]. However, as in the present case, the prenatal ultrasound findings of sacrococcygeal teratoma and spinal bifida aperta can be very similar. For example, Yu et al. reported a case of myelocystocele that was prenatally diagnosed as sacrococcygeal teratoma [6].

The treatment options for sacrococcygeal meningocele depend on several factors, including the size, location, and presence of associated anomalies [1-2]. Excision and primary closure are suitable for small to medium-sized meningoceles without bony defects or other anomalies. Excision and closure with a flap or muscle flap are useful for large meningoceles with limited tissue. Excision and staged closure are appropriate for very large meningoceles with significant bony defects [3-5]. A combined approach involving neurosurgical and plastic surgery teams is necessary for cases with associated spinal dysraphism or other neural anomalies. Ultimately, the choice of technique depends on multiple factors, including the surgeon's experience.

## Conclusions

In conclusion, we presented a case of a sacrococcygeal meningocele in a newborn who was successfully treated with surgical intervention, resulting in a favorable prognosis. Our case underscores the challenge of distinguishing between sacrococcygeal teratoma and meningocele based on clinical presentation and antenatal ultrasound findings. Accurate preoperative diagnosis is essential for optimal surgical planning and management.
